# Supplementary material for: Area extraction and spatiotemporal characteristics of winter wheat–summer maize in Shandong Province using NDVI time series
Source: PLoS One. 2019 Dec 12;14(12):e0226508. doi: 10.1371/journal.pone.0226508 (PMC6907822; doi:10.1371/journal.pone.0226508)
Supplement: Attachment — Submitted filename: Response to Reviewers.docx [file pone.0226508.s001.docx]

Dear Editor:

Thank you for handling our manuscript entitled “Area extraction and spatiotemporal characteristics of winter wheat–summer maize in Shandong Province using NDVI time series” (No.: PONE-D-19-20112). We appreciate the comments from the reviewers, which helped to improve the manuscript significantly. In the following section, we explain in detail how we responded to each of the comments.

**Responses to Journal' comments:**

**Comment #1:**

Ensure that your manuscript meets PLOS ONE's style requirements, including those for file naming.

**Response #1:**

We are very grateful for your comments of this research. We have adjusted the article format according to PLOS ONE's style requirements. If there are still problems, we would very much like to receive your guidance and seriously revise them.

**Comment #2:**

We note that Figures in your submission contain map/satellite images which may be copyrighted.

**Response #2:**

As suggested by the editor, we checked the images in our submission. The images in our submission were all produced by using MODIS data products, and we used these images to make figures. The LAADS DAAC Data Use Policies and Copyright(<https://modaps.modaps.eosdis.nasa.gov/services/faq/LAADS_Data-Use_Citation_Policies.pdf>) shows that these data products are not copyrighted, but they request that the paper need to include an acknowledgment and citation. We added citation and acknowledgment as requested by copyright as follows:

Page 7 Line 126-128: The citation was added as “We used the vegetation indices of 16-day composites from Terra/MODIS (MOD13Q1, h27v05) [28] and Aqua/MODIS (MYD13Q1, h27v05) [29] at a spatial resolution of 250 m.”

Page 24 Line 435-439: The acknowledgment was added as “The MODIS/Terra Vegetation Indices 16-Day L3 Global 250m SIN Grid and MODIS/Aqua Vegetation Indices 16-Day L3 Global 250m SIN Grid datasets were acquired from the Level-1 and Atmosphere Archive & Distribution System (LAADS) Distributed Active Archive Center (DAAC) located at the Goddard Space Flight Center in Greenbelt, Maryland (<https://ladsweb.nascom.nasa.gov/>).”

**Comment #3:**

Please include captions for your Supporting Information files at the end of your manuscript, and update any in-text citations to match accordingly.

**Response #3:**

As suggested by the editor, the Supporting Information was added at the end of the manuscript. Supporting Information only contains funding information, which we did not quote in the article.

**Responses to Reviewers' comments:**

**Reviewer #1:**

**Comment #1:**

The methodological novelty is not well articulated in the abstract and introduction.

**Response #1:**

We are very appreciated with these suggestions by the reviewers and agree with this. We made some corresponding changes in the manuscript as follows:

In the abstract, we added a description of the method and the advantages.

Page 2 Line 22-25: The sentence was added as “Therefore, we mapped a spatial distribution of the winter wheat and summer maize, using the time series data pre-processing synthesis and phenology curve random forest classification methods. Through precision analysis, we obtained satisfactory results, which provided a straightforward and efficient method to monitor the winter wheat and summer maize.”

In the introduction, we highlighted the novelty by emphasizing time series data production and phenological classification methods. We increased the citation of phenology classification (Page 3 Line 46-49 and Page 3 Line 59-63) to emphasize the importance of phenology classification in remote sensing classification. Then we added the shortcomings of phenology research.

Page 4 Line 65-67: The sentence was added as “For how to use remote sensing data to indicate phenological information, the current research is mainly focused on single satellite data, but less on double satellite data. In addition, for example, to improve the quality of data set and to better characterize the phenological curve need to be studied.”

In the section (Page 4 Line 82-89), we had analyzed the aspects that need to be mined. So we add a description of methods to achieve objectives at the end of introduction.

Page 5 Line 95-98: The sentence was added as “The objective of this study is to be able to quickly draw crop maps with high precision. This method makes full use of the advantages of time series data to reflect the phenological characteristics. Combined with the wide coverage of MODIS data, this method can better achieve this objective.”

**Comment #2:**

The description of the study area can be reduced.

**Response #2:**

As suggested by the reviewer, the description of the study area had been reduced. The details are as follows:

Page 5 Line 102: We deleted the description of latitude and longitude, which can be obtained from the figure.

Page 5 Line 104-105 and Page 5 Line 106-108: We also reduced the description of terrain and climate which were not related to phenology or spatial distribution of the crop.

Page 5 Line 101-112: There were some other minor modifications, the revised description of the study area as “Shandong Province is located in the North China Plain between the lower reaches of the Yellow River and eastern coast (Fig 1). Shandong Province consists of two parts, i.e., the peninsula and inland region, with a land and cultivated areas of 15.7 of 7.52 Mha, respectively. The study area can be divided into three landforms: hilly areas of the middle southern region, western plains, and eastern hilly regions. The province has an average of 2,290–2,890 hours of sunshine per year, such that the heating conditions can conform to the needs of two crop rotations per year. There are currently both double- and single-cropping systems in Shandong Province [25], where the main grain crops are wheat and maize.”

**Comment #3:**

Paragraphs in the conclusion section should not be numbered.

**Response #3:**

As suggested by the reviewer, the paragraph numbers in the conclusion were deleted.

**Comment #4:**

Define the mtry and ntry parameters used in running the RF model.

**Response #4:**

We consider it is necessary to give the define about the parameters. In this study, we used the random forest module provided by ENMAP Box V2.2, which can examine information on RFC models. The model window contains information on the training data used and out-of-bag-error (OOB). Number of trees is confirmed by observing OOB error rate. ENMAP also provides the function to determine the number of randomly selected features to compute the best split point or set this number manually. After testing, we choose the parameter of the function as the square root of the number of all features.

Page 9 Line 186-188: As suggested by the reviewer, the parameters were defined as “The value for the number of trees used was 100. The function to determine the number of randomly selected features was the square root of the number of all features.”

**Comment #5:**

Proper English language editing is also required.

**Response #5:**

As suggested by the reviewer, we invited Editage (www.editage.cn) language editing service to make a comprehensive revision of the article language.

|  | **DOGCH_1_3** Do it again.  Filter service items: Quality Polish  Delivery time:2019/10/24(Thursday)17:03(CST) |
| --- | --- |

**Reviewer #2:**

**Comment #1:**

The importance of food crops such as maize and wheat is well known, and there is no need for motivation on that, what is lacking in the introduction is the motivation why is it so important to study the phenology of these food crops.

**Response #1:**

As suggested by the reviewer, we added the importance description of Crop Phenology in the introduction. The content of Crop Phenology information and classification accuracy was added in the discussion.

Page 3 Line 46-49: The sentence was added in the introduction as “Crop phenology is an appropriate growth and development rhythm formed by an organism's long-term adaptation to temperature conditions. Crop phenological information is not only the basis for agricultural production decisions but also an important parameter for crop simulation models [12]. Understanding phenological changes in crop monitoring and ecological prediction is highly important.”

Page 20 Line 361-363: The sentence was added in the discussion as “The results show that the phenological characteristic of the winter wheat–summer maize was significantly different from that of other land cover, which can be used as a significant indicator in crop remote sensing monitoring.”

Page 20 Line 365-379: The sentence was revised in the discussion as “At the same time, the time series data contains not only the spatial distribution characteristics of crops, but also the temporal characteristic, which is equivalent to adding a significant phenological indicator in the extraction. More information means higher classification accuracy. With the accumulation of remote sensing data, temporal features should be taken as the basic important parameters to participate in remote sensing crop monitoring.”

**Comment #2:**

In the introduction, the authors introduce MODIS as a medium spatial resolution sensor (Page 3 line 54) and a few moments later, MODIS is now a low spatial resolution sensor (Page 3 line 58), please rectify.

**Response #2:**

Page 2 Line 43: We are sorry for this mistake. As suggested by the reviewer, the mistake has been fixed.

**Comment #3:**

Also explain how do phenology metrics solve the problem of low spatial resolution?

**Response #3:**

We added a citation about phenology to improve the accuracy of remote sensing classification, and explained the problems of remote sensing classification that phenology index mainly solves.

Page 3 Line 52-55: As suggested by the reviewer, the citation sentence was added as “Phenological characteristics can provide information other than spatial spectral characteristics. As different vegetation has unique phenological characteristics, phenological characteristics are considered a remote sensing method whose application can improve the accuracy of the vegetation classification [14].”

Page 3 Line 61-63: As suggested by the reviewer, the sentence was added as “Based on these phenological differences, remote sensing extraction can resolve the foreign body homology problem and improve the classification accuracy.”

**Comment #4:**

It is not clear which phenological metrics were used and why, how were these phenological metrics extracted, which algorithm (s) were used? All this information should be clear in the methods section.

**Response #4:**

As suggested by the reviewer, we added the content related to phenology parameter setting in the method section. First, we used phenology curve to confirm the training data label. Second, the RF model uses the NDVI time series data, and the data of each time node of the crop is used as the input parameter to participate in the classification.

Page 9 Line 171-175: The sentence was added as “Based on these categories, the category sampling points were generated. The data category labels were further determined based on the high-resolution satellite images from Google Earth. The sampling point used the spatial analysis to extract the timing curve from the MODIS time series. We calculated the mean and standard deviation of the curve by category and plotted it. The temporal curve shows the phenology of the vegetation above this category. We used the curve as a sign of interpretation.”

Page 9 Line 185-189: The sentence was added as “The First RF Classification (RFC) model was parameterized using training points (i.e., the phenological features of ground objects) for training and internal validation. The value for the number of trees used was 100. The function to determine the number of randomly selected features was the square root of the number of all features. In a second step, the phenology model was used to perform the MODIS time series.”

**Comment #5:**

Figure 4 shows NDVI temporal curves for five categories, the main objective of this study was to extract spatiotemporal characteristics of winter wheat–summer maize, therefore l do not see the relevance of the other categories presented here. The description citing figure 4 is using months but the graphical presentation on figure 4 is using days, for the sake of consistency, l advice the authors to use either days or moths for both.

**Response #5:**

As suggested by the reviewer, we modified figure 4 to remove categories except the class of winter-summer maize. The figure was modified as:

Fig 4. NDVI temporal curves of the winter–summer maize.

As suggested by the reviewer, we have unified the description of the date.

Page 11 Line 217-219: The sentence was revised as “The first peak occurs between days 60 and 170 during the winter wheat growth cycle while the second peak appears between days 180 and 270 during the summer maize growth cycle, which is consistent with the growth cycle of winter wheat–summer maize.”

**Comment #6:**

In the discussion, the authors claim that they have developed a tool for extracting spatiotemporal distribution information from large-scale winter wheat–summer maize planting from the MODIS-NDVI time series. However, the ‘newly developed’ tool was not mentioned, please clarify.

**Response #6:**

Page 19 Line 357: As suggested by the reviewer, the sentence was revised as “This study extracted spatiotemporal distribution information from large-scale winter wheat–summer maize planting from the MODIS-NDVI time series dataset.”

**Comment #7:**

Page 3 line 50: Give examples of the high spatial resolution images you are referring to.

**Response #7:**

Page 2 Line 37-39: As suggested by the reviewer, the sentence was revised as “such as the landsat and SPOT images, to map winter groundcover on agricultural fields [7]. RapidEye images were used to derive training data for the characterization of time-series Landsat data [8].”

**Comment #8:**

Page 4 line 66-68: The works of Li et al. [15] do not add value to the current work if their research findings are not reported, mentioning what their research focus only is not good enough

**Response #8:**

Page 3 Line 59-63: As suggested by the reviewer, the sentence was revised as “Li et al. [16] extracted crops, discussed the spatiotemporal patterns of the crop phenological period/planting system in north China, and verified that the quantity distribution and spatial distribution of crop phenological characteristics were significantly different in the different growing seasons. Based on these phenological differences, remote sensing extraction can resolve the foreign body homology problem and improve the classification accuracy.”

**Comment #9:**

Page 8: Under the ‘smooth processing of time series’ sections please provide a graphical presentation of raw and smoothed NDVI temporal data

**Response #9:**

As suggested by the reviewer, we adjusted Fig 3 to include the comparison of raw and smoothed NDVI temporal data as:

**Fig 3. A comparison of the temporal data processing.**

Note: (a) and (b) are handled by QA comparisons with the raw data while (c) and (d) are handled by HANTS comparisons with raw data.

**Comment #10:**

Page 11 line 214: ‘land types’ do you mean land cover types or land use types?

**Response #10:**

Page 11 Line 217: In this study, we refer to land cover types, we reunified the description to ‘land cover type’ (Page 9 Line 180). Another sentence containing land cover (Page 11 Line 217) has been modified because of **Comment #5**.

**Comment #11:**

Page 20 line 367: Replace ‘good agreement with reality’ with ‘good agreement with field/ground observation/measurements.

**Response #11:**

Page 18 Line 359: We followed the suggestion, the ‘reality’ was revised as ‘field’.
