# Supplementary material for: Area extraction and spatiotemporal characteristics of winter wheat–summer maize in Shandong Province using NDVI time series
Source: PLoS One. 2019 Dec 12;14(12):e0226508. doi: 10.1371/journal.pone.0226508 (PMC6907822; doi:10.1371/journal.pone.0226508)
Supplement: Attachment — Submitted filename: Response to Reviewers.docx [file pone.0226508.s002.docx]

Dear Editor:

Thank you for handling our manuscript entitled “Area extraction and spatiotemporal characteristics of winter wheat–summer maize in Shandong Province using NDVI time series” (No.: PONE-D-19-20112). We sincerely appreciate your letter and reviewers for their valuable comments and feel encouraged by their feedback. We thank you gratefully and all the reviewers for their valuable comments and suggestions that greatly improved our manuscript. Our point-by-point responses to the reviewers' comments are given as follows.

**Responses to Reviewers' comments:**

**Reviewer #2:**

**Comment #1:**

Figure 4 does not have a legend, what is the red dotted and the black line representing?

**Response #1:**

We are very sorry that we missed the legend. We have added the legend to Fig 4 as:

**Fig 4. NDVI temporal curves of the winter**–**summer maize.** (DOY: day of year)

**Comment #2:**

Page 10 line 191: I suggest you change the section ‘Accuracy validation’ to accuracy assessment.

**Response #2:**

As suggested by the reviewer, we have replaced “Accuracy validation” by “accuracy assessment”. (Page 9 Line 171 and 177)

**Comment #3:**

Page 10 line 194: Consider rephrasing the sentence which begins with ‘To evaluate the comprehensiveness and objectivity of..’ since the previous sentence begins exactly the same.

**Response #3:**

We agree. The second sentence is not necessary, we have deleted this statement.

**Comment #4:**

Page 10 line 196-197: ‘high-resolution images from google earth’ Which resolution are you referring to? Spatial, spectral, temporal?

**Response #4:**

As suggested by the reviewer, we revised the sentences as “high spatial resolution images from google earth”.

**Comment #5:**

Page 11 line 216: The first heading on the results section needs attention, which remote sensing ‘approaches’ are you referring to? The authors should specify the remote sensing approaches they used.

**Response #5:**

There is an error in our expression. What we mean is NDVI time series data profile. We have revised the first heading.

We hope that you can satisfy the above responses. If you have any questions, please let us know so as to improve our manuscript. Thank you for your suggestions.
